# Dexamethasone inhibits activation of monocytes/macrophages in a milieu rich in 27-oxygenated cholesterol

**DOI:** 10.1371/journal.pone.0189643

**Published:** 2017-12-13

**Authors:** Bo-Young Kim, Yonghae Son, Jeonga Lee, Jeongyoon Choi, Chi Dae Kim, Sun Sik Bae, Seong-Kug Eo, Koanhoi Kim

**Affiliations:** 1 Department of Pharmacology, Pusan National University—School of Medicine, Yangsan, Gyeongnam, Republic of Korea; 2 College of Veterinary Medicine and Bio-Safety Research Institute, Chonbuk National University, Iksan, Jeonbuk, Republic of Korea; Universite Clermont Auvergne, FRANCE

## Abstract

Molecular mechanisms underlying the decreased number of macrophages and T cells in the arteries of cholesterol-fed-rabbits following dexamethasone administration are unknown. We investigated the possibility that dexamethasone could affect activation of monocytic cells induced by oxygenated derivatives of cholesterol (oxysterols) using THP-1 monocyte/macrophage cells. 27-Hydroxycholesterol (27OHChol), an oxysterol elevated with hypercholesterolemia, enhanced production of CCL2, known as MCP1, chemokine from monocytes/macrophages and migration of the monocytic cells, but the CCL2 production and the cell migration were reduced by treatment with dexamethasone. Dexamethasone inhibited superproduction of CCL2 induced by 27OHChol plus LPS and attenuated transcription of matrix metalloproteinase 9 as well as secretion of its active gene product induced by 27OHChol. The drug downregulated cellular and surface levels of CD14 and blocked release of soluble CD14 without altering transcription of the gene. Dexamethasone also inhibited expression and phosphorylation of the NF-κB p65 subunit enhanced by 27OHChol. Collectively, these results indicate that dexamethasone inhibits activation of monocytes/macrophages in response to 27OHChol, thereby leading to decreased migration of inflammatory cells in milieu rich in oxygenated derivatives of cholesterol.

## Introduction

Dexamethasone, a synthetic glucocorticoid with anti-inflammatory and immunosuppressant effects, is used to treat many inflammatory conditions, including allergic disorders, skin conditions, ulcerative colitis, arthritis, lupus, psoriasis, and breathing disorders [1]. However, prolonged corticosteroid therapy can induce or exacerbate coronary risk factors, such as hypertension, hypercholesterolemia, hypertriglyceridemia, and impairment of glucose tolerance [2–4]. In animal experiments, dexamethasone has been reported to inhibit cell migration, thereby reducing development of atherosclerosis. Administration of dexamethasone results in a decreased number of immune cells, such as macrophages and T lymphocytes, in the intima of cholesterol-fed rabbits [5–7]. These findings suggest that inhibitory effects of the drug on cellular responses to high cholesterol may occur, but it is unknown how it exerts such effects in a milieu rich in cholesterol molecules.

Cholesterol molecules accumulate in the artery where they undergo oxidation to oxysterols, the oxygenated derivatives of cholesterol. Oxysterols are biologically active molecules affecting multiple types of cells. Oxysterols oxidized at C7 of cholesterol differentially activate human umbilical venous endothelial cells and play a key role in apoptosis and IL-1β secretion [8]. Oxysterol-induced IL-8 secretion is a calcium-dependent phenomenon leading to the IL-8 gene activation via the MEK/ERK1/2 and AP-1 (c-fos) [9]. 27-Hydroxycholesterol (27OHChol), the most abundant oxysterol in atherosclerotic arteries and in sera of patients suffering hypercholesterolemia, is synthesized by sterol 27-hydroxylase or formed by non-enzymatic oxidation [10, 11], and the levels of 27OHChol are two orders of magnitude higher than circulating levels in the arteries [12]. 27OHChol promotes inflammatory process *in vivo* [13], presumably via activation of monocytic cells. The oxysterol increases expression of pattern recognition receptors and enhances migration of immune cells by inducing secretion of chemoattractants such as CCL2, which is widely known as MCP1, CCL3 and CCL4 from monocyte/macrophage cells [14–17]. These findings indicate that 27OHCHol is a key oxygenated cholesterol derivative that activates monocytic cells.

We investigated whether dexamethasone affects monocytes/macrophages activation in a milieu rich in 27-oxygenated derivative of cholesterol to understand the molecular mechanisms underlying its anti-migration effect in cholesterol-fed animals.

## Materials and methods

### Reagents

Dexamethasone was acquired from Enzo Life Sciences (Farmingdale, NY, USA). 27OHChol and antibodies against CD14, p65, phosphorylated p65, and β-actin were obtained from Santa Cruz Biotechnology Inc. (Santa Cruz, CA, USA). Lipopolysaccharide (LPS-EK) from *Escherichia coli K12* was purchased from InvivoGen (San Diego, CA, USA).

### Cell treatments

Human monocytic THP-1 cell line was purchased from the American Type Culture Collection (ATCC, Manassas, VA, USA) and maintained in 10% FBS-supplemented RPMI 1640 medium. THP-1 cells (2.5 × 10^5^ cells/ml) were serum-starved by incubating overnight in RPMI medium supplemented with 0.1% BSA (endotoxin-free), and the serum-starved cells were treated with 27OHChol in the presence of indicated concentrations of dexamethasone. 27OHChol and dexamethasone were dissolved in ethanol and dimethyl sulfoxide, respectively. In experiment of CCL2 superinduction, serum-starved THP-1 cells were cultured for 24 h with 27OHChol in the presence of dexamethasone, followed by stimulation for 9 h with LPS (100 ng/ml) dissolved in endotoxin-free water.

### Reverse transcription (RT)—Real-time polymerase chain reaction (PCR)

Total RNA was reverse-transcribed for 1 h at 42°C with Moloney murine leukemia virus reverse transcriptase. Real-time PCR was performed in triplicate using a LightCycler^®^ 96 Real-Time PCR System (Roche, Germany). Each 20 μl reaction consisted of 10 μl of SYBR Green Master Mix and 2 μl of 10 pM forward and reverse primers of the gene to be quantified. The thermal cycling conditions consisted of 95°C for 10 min, followed by 45 cycles of 95°C for 10 sec, 50°C for 10 sec, and 72°C for 10 sec. The relative expression of each gene was calculated as the ratio to GAPDH as a housekeeping gene using the LightCycler^®^ 96 software (Version 1.1.0.1320, Roche, Germany). The primers were as follows: CCL2, 5′-CAGCCAGATGCAATCAATGCC-3′ (forward) and 5′-TGGAATCCTGAACCCACTTCT-3′ (reverse); and matrix metalloprotease-9 (MMP-9), 5′-GCACGACGTCTTCCAGTACC-3′ (forward) and 5′-CAGGATGTCATAGGTCACGTAGC-3′(reverse); CD14, 5′-ACGCCAGAACCTTGTGAGC-3′ (forward) and 5′-GCATGGATCTCCACCTCTACTG-3′ (reverse), p65: 5′-ATCCCATCTTTGACAATCGTGC-3′ (forward) and 5′-CTGGTCCCGTGAAATACACCTC-3′ (reverse); GAPDH, 5′-GAAGGTGAAGGTCGGAGT-3′ (forward) and 5′-GAAGATGGTGATGGGATTTC-3′ (reverse).

### Chemotaxis assay

Migration of THP-1 cells was measured using Transwell Permeable Supports (Costar, Cambridge, MA, USA) as previously described [16]. Cells (5 × 10^5^ cells in 100 μL of 0.1% BSA) were loaded into the top chamber of 5-μm-pore polycarbonate transwell inserts. Transwell chambers were inserted into wells filled with a supernatant isolated from THP-1 cells treated with 27OHChol with or without dexamethasone. After incubation for 3 h at 37°C, the number of cells that migrated to the bottom chamber was counted using a Vi-Cell cell counter (Beckman Coulter, Inc. Brea, CA, USA).

### MMP-9 gelatinolytic activity in supernatants

Supernatants isolated from cultured THP-1 cells were collected and concentrated 30-fold using Vivaspin 2 Centricon as previously described [18]. The concentrated medium was then electrophoretically separated onto an 8% polyacrylamide gel containing 0.15% gelatin. After electrophoresis, the gel was washed, activated, and stained with 0.2% Coomassie brilliant blue R-250 prior to destaining. Clear zones against the blue background indicated gelatinolytic activity.

### Flow cytometric analysis

THP-1 cells were harvested by centrifugation and incubated for 40 min with anti-CD14 antibody conjugated with a green fluorescent dye (Santa Cruz Biotechnology Inc.) at 4°C. After washing twice with PBS, cells were resuspended in 1% paraformaldehyde in phosphate-buffered saline (PBS). Fluorescence was analyzed by flow cytometry.

### Enzyme-linked immunosorbent assay

The levels of CCL2, sCD14, and MMP-9 secreted into the culture media were determined using commercially available enzyme-linked immunosorbent assay (ELISA) kits according to the manufacturer's instructions (R&D Systems, Minneapolis, MN, USA).

### Western blot analysis

Cell lysates were separated by 10% SDS-PAGE followed by transfer to nitrocellulose membranes. After blocking for 1 h in 1% skim milk (in TBS containing 0.05% Tween-20), membranes were incubated with primary antibodies against CD14, phosphorylated p65, p65 subunit or β-actin at 4°C overnight. After three washes with TBS-T, membranes were incubated for 1 h with HRP-conjugated secondary antibodies at room temperature. Bands were detected using chemiluminescent detection reagents.

### Statistical analysis

One-way ANOVA followed by Dunnett's multiple comparison tests was performed using PRISM (version 5.0) (GraphPad Software Inc., San Diego, CA, USA). Null hypotheses of no difference were rejected if p-values were less than 0.05.

## Results

### Inhibitory effects on expression of CCL2 and migration of monocytic cells

**Fig 1:**
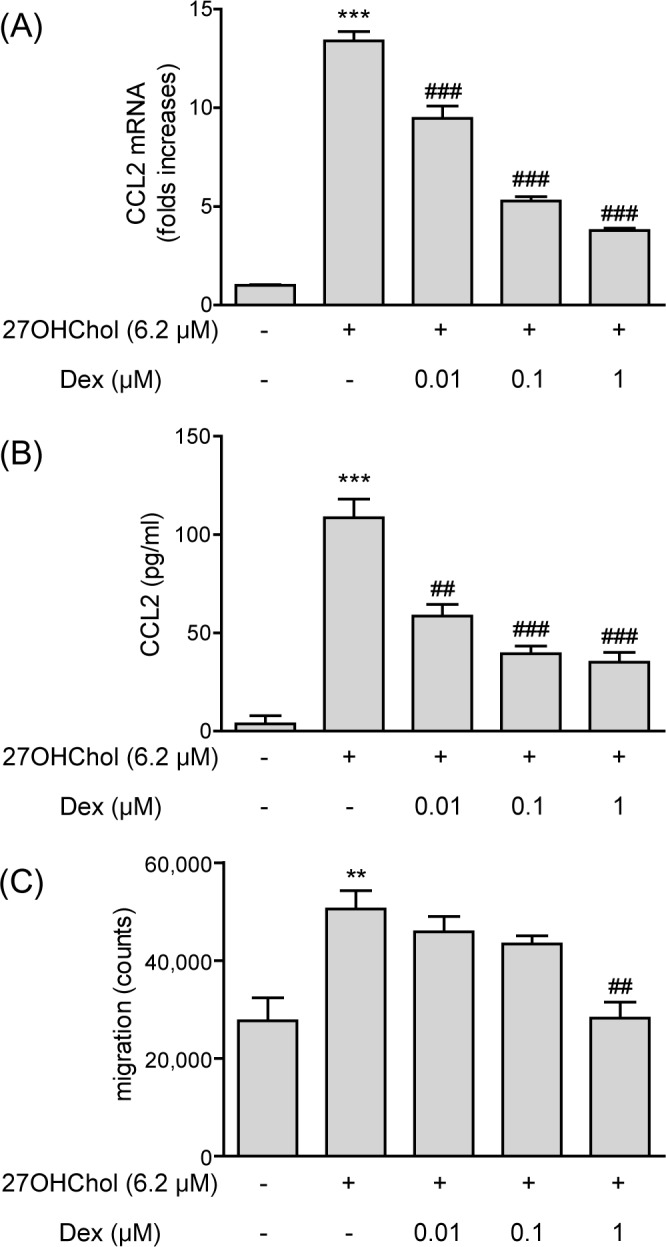
Effects of dexamethasone (Dex) on CCL2 expression and monocytic cell migration. (A) Levels of CCL2 transcript were assessed by real-time PCR. The y-axis values represent increases in CCL2 mRNA levels normalized to GAPDH levels, relative to that of the non-treated THP-1 cells (control). Data are expressed as the means ± SD (n = 3 replicates for each group). (B) Culture media were isolated, and the levels of CCL2 protein in the media were measured by ELISA. Data are expressed as the means ± SD (n = 3 replicates for each group). (C) Monocytic cells were exposed to the conditioned media isolated above, and migration of monocytic cells was measured by chemotaxis assay. Data are expressed as the means ± SD (n = 3 replicates for each group). The results shown are the representative of three independent experiments. *** P < 0.001 vs. control; ** P < 0.01 vs. control; ### P < 0.001 vs. 27OHChol; ## P < 0.01 vs. 27OHChol.

We investigated whether dexamethasone affected expression of CCL2 chemokine. Transcription of the CCL2 gene was induced in the presence of 27OHChol, and the induction was attenuated by treatment with dexamethasone in a dose-dependent manner (Fig 1A). CCL2 transcript levels were increased by 13.4-fold in the presence of 27OHChol, but reduced to 9.5-, 5.3-, and 3.8-folds by treatment with 0.01, 0.1, and 1 μM of dexamethasone, respectively. Dexamethasone influenced CCL2 secretion in a pattern similar to that observed with transcription of the CCL2 gene (Fig 1B). The amount of CCL2 secreted from THP-1 cells increased significantly in the presence of 27OHChol compared with that secreted from unstimulated THP-1 cells. The levels of CCL2 secreted from 27OHCHol-treated THP-1 cells were dose-dependently reduced by treatment with dexamethasone. The reduction was not due to cytotoxicity because dexamethasone did not affect cell viability (S1 Fig).

We conducted a chemotaxis assay to determine if dexamethasone alters migration of monocytic cells (Fig 1C). Migration of monocytic cells was blocked by treatment with high concentrations of dexamethasone. Monocytic cell migration was increased in response to the supernatant isolated from cells stimulated with 27OHChol. The migration was slightly reduced by treatment with 0.01 and 0.1 μM of dexamethasone and even further reduced to basal levels by 1 μM dexamethasone. Overall, these results indicate that dexamethasone inhibited CCL2 expression and monocytic cell migration induced by 27OHChol.

### Inhibition of CCL2 superinduction

**Fig 2:**
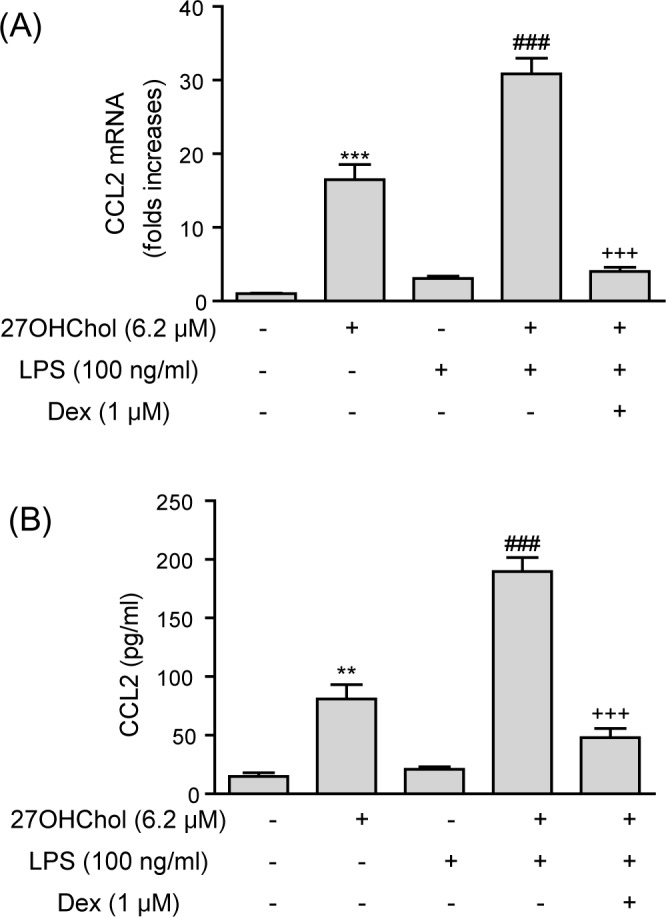
Effects of dexamethasone (Dex) on superproduction of CCL2. (A) Levels of CCL2 transcript were assessed by real-time PCR. Data are expressed as the means ± SD (n = 3 replicates for each group). (B) Culture media were isolated, and the amount of CCL2 protein secreted into the media was measured by ELISA. Data are expressed as the means ± SD (n = 3 replicates for each group). The results shown are the representative of three independent experiments. *** P < 0.001 vs. control; ** P < 0.01 vs. control; ### P < 0.001 vs. 27OHChol; +++ P < 0.001 vs. 27OHChol plus LPS.

Expression of CCL2 is super-induced when LPS is added to monocytic cells stimulated with 27OHChol [18]. We investigated whether dexamethasone influences super-induction of CCL2. Transcript levels of the CCL2 gene were increased by 16.5- and 3.4-folds in the presence of 27OHChol and LPS, respectively (Fig 2A). The addition of LPS to 27OHChol-stimulated cells resulted in a 30.8-fold elevation in CCL2 transcript levels, but the elevation was blocked by treatment with dexamethasone. Dexamethasone influenced secretion of CCL2 in a pattern similar to that of transcription (Fig 2B). 27OHChol increased secretion of CCL2, and the addition of LPS to 27OHChol-stimulated cells resulted in further enhanced secretion of CCL2. The enhanced CCL2 secretion was significantly attenuated by treatment with dexamethasone. These results indicate that dexamethasone suppressed super-production of CCL2 induced by LPS in combination with 27OHChol.

### Downregulation of 27OHChol-induced expression of CD14 at the protein level

**Fig 3:**
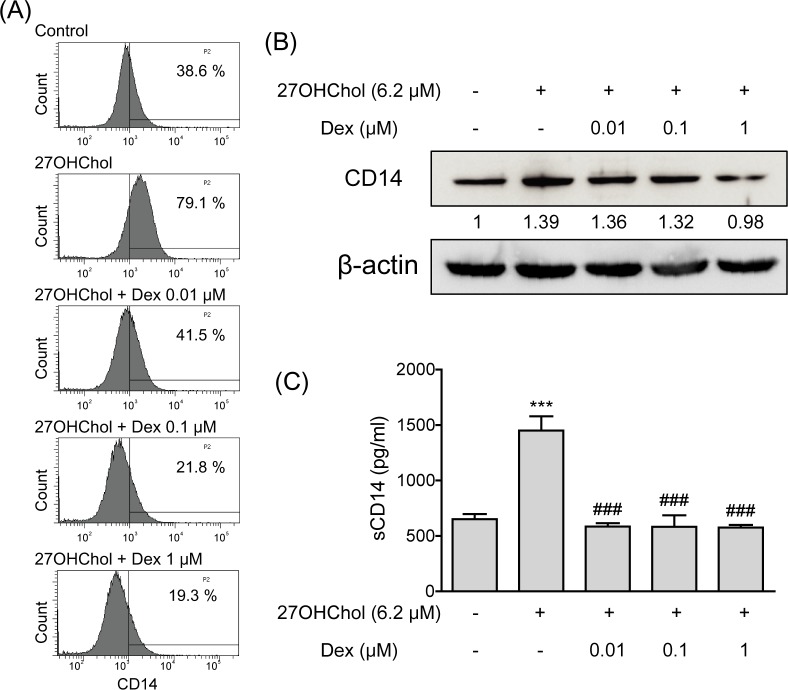
Attenuated production of CD14 protein by dexamethasone (Dex). (A) CD14 on surface of THP-1 cells was labeled with fluorescein isothiocyanate (FITC), and the fluorescence was analyzed by flow cytometry. The x axis represents relative fluorescence intensity of the labeled cells, and the y axis represents the number of cells found at each fluorescence level. (B) Cell extracts were obtained from THP-1 cells after treatment with or without 27OHChol and dexamethasone, followed by Western blot analysis to detect CD14 and β-actin. Data are representative of three independent experiments. (C) Culture media were isolated, and the amount of CD14 protein secreted into the media was measured by ELISA. Data are expressed as the means ± SD (n = 3 replicates for each group). The results shown are the representative of three independent experiments. *** P < 0.001 vs. control; ### P < 0.001 vs. 27OHChol.

Because LPS responses depend on the expression of membrane CD14 (mCD14) by monocytic cells [19], we investigated whether dexamethasone downregulates mCD14 on the surface of monocytic cells. The percentage of cells expressing mCD14 was increased from 38.6% to 79.1% in the presence of 27OHChol, but it was reduced to 41.5%, 21.8%, and 19.3% by treatment with 0.01, 0.1, and 1 μM of dexamethasone, respectively (Fig 3A). We also evaluated the effects of dexamethasone on CD14 protein via Western blot analysis (Fig 3B), and found that the level of total CD14 protein was increased in the presence of 27OHChol. However, the expression of CD14 protein was reduced by treatment with 0.01 and 0.1 μM of dexamethasone and further decreased to below the basal level by 1 μM dexamethasone. We also investigated whether dexamethasone affected transcription of CD14. Transcript levels of the CD14 were elevated in the presence of 27OHChol, and the elevation was not decreased by treatment with dexamethasone (S2 Fig).

A soluble form of the CD14 molecule (sCD14) is capable of activating monocytic cells independently of mCD14 [20]. Therefore, we determined whether dexamethasone alters generation of sCD14. THP-1 cells released a basal level of sCD14 of 651.2 ± 79.7 pg/ml to the media, and the level increased significantly to 1450.8 ± 222.2 pg/ml in the presence of 27OHChol (Fig 3C). However, the increased release of sCD14 was blocked by dexamethasone. The amounts of sCD14 released from 27OHChol-treated THP-1 cells were reduced to the basal level by treatment with 0.01, 0.1 or 1 μM of dexamethasone. These findings indicate that dexamethasone suppresses expression of CD14 protein and production of sCD14.

### Attenuation of 27OHChol-induced expression of MMP-9

**Fig 4:**
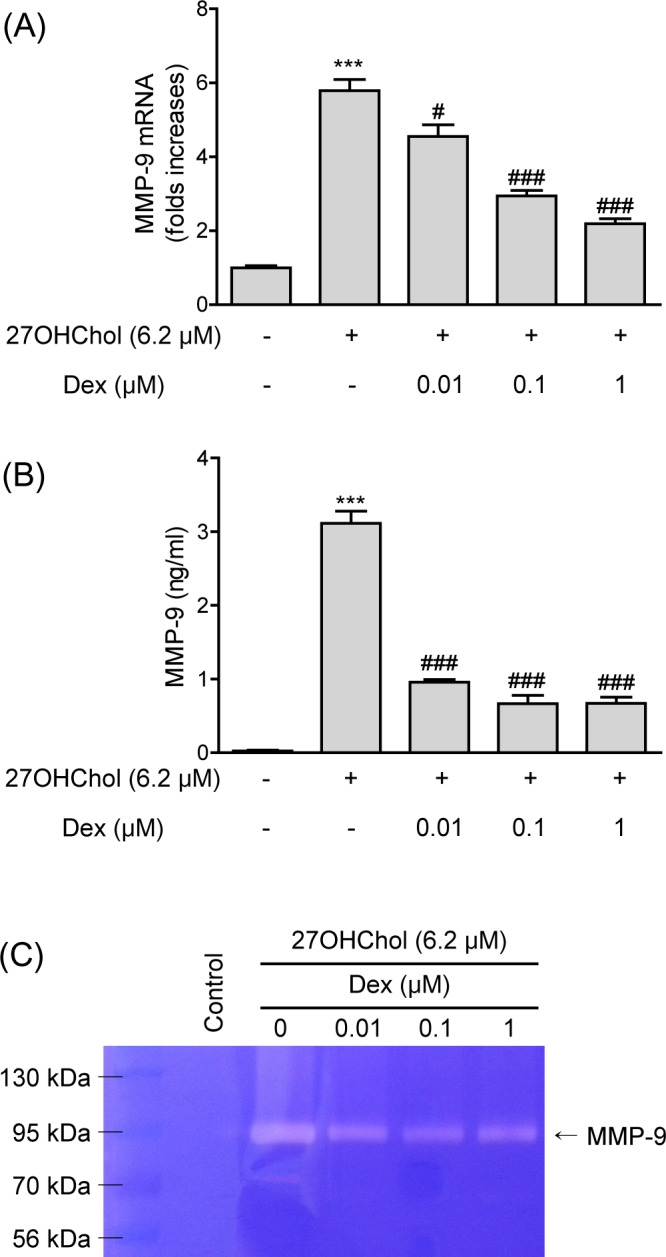
Effects of dexamethasone (Dex) on expression of MMP-9 induced by 27OHChol. (A) Levels of MMP-9 transcript were assessed by real-time PCR. Data are expressed as the means ± SD (n = 3 replicates for each group). (B) Culture media were isolated, and the levels of MMP-9 in the media were measured by ELISA. Data are expressed as the means ± SD (n = 3 replicates for each group). The results shown are the representative of three independent experiments. *** P < 0.001 vs. control; ### P < 0.001 vs. 27OHChol; # P < 0.05 vs. 27OHChol. (C) The activity of MMP-9 secreted by cells was assessed by gelatin zymography. Control THP-1 cells were cultured for 48 h in the medium alone. Data are representative of three independent experiments.

Proteolytic cleavage of mCD14 by MMP-9 is one of the mechanisms generating sCD14 [21]. Therefore, we examined the effects of dexamethasone on MMP-9 expression. Transcript levels of the MMP-9 gene were increased by 5.8-fold in the presence of 27OHChol. The increase, however, was reduced to 4.6-, 2.9-, and 2.2-folds in response to treatment with 0.01, 0.1, and 1 μM of dexamethasone, respectively (Fig 4A).

We determined the effects of dexamethasone on production of MMP-9 protein by evaluating the total (latent and active) levels of MMP-9 secreted into the medium (Fig 4B). THP-1 cells secreted a low basal level of MMP-9, and the amount of secreted MMP-9 protein was significantly increased to 3.1 ± 0.3 ng/ml in the presence of 27OHChol, which was reduced to 1.0 ± 0.1, 0.7 ± 0.1, and 0.7 ± 0.09 ng/ml by treatment with 0.01, 0.1, and 1 μM of dexamethasone, respectively. We next examined whether dexamethasone affected the activity of MMP-9 by zymography. Stimulation of THP-1 cells with 27OHChol resulted in increased MMP-9 activity in supernatant, but this increase in activity was attenuated by treatment with dexamethasone (Fig 4C). These results mean that dexamethasone inhibits the monocytic cell expression of MMP-9 induced by 27OHCHol at the mRNA and protein levels.

### Suppression of NF-κB p65 expression

**Fig 5:**
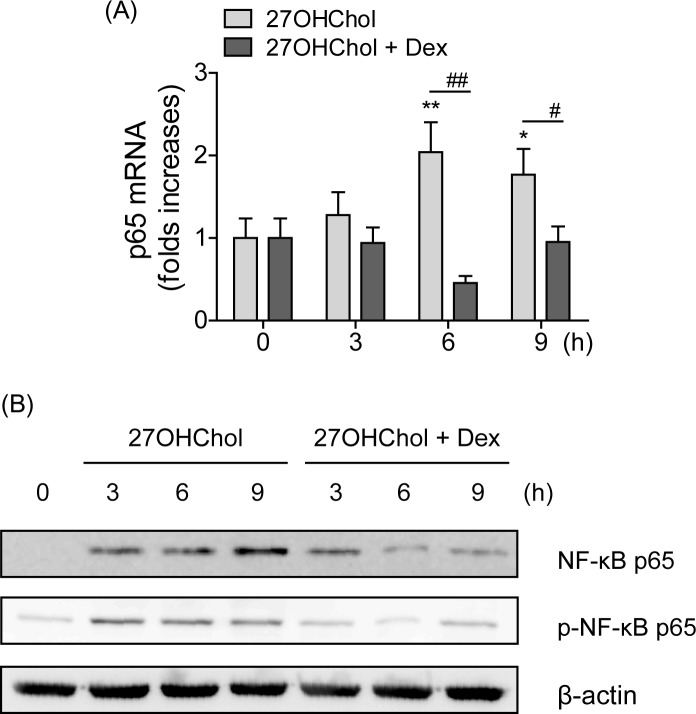
Effects of dexamethasone on p65 subunit expression. Serum-starved THP-1 cells (2.5 × 10^5^ cells/ml) were cultured for the indicated time periods with 27OHChol (6.2 μM) in the absence or presence of 1 μM dexamethasone (Dex). (A) Levels of p65 transcript were assessed by real-time PCR. The results shown are the representative of three independent experiments. Data are expressed as the means ± SD (n = 3 replicates for each group). Statistical differences among treatment time periods were evaluated by One-way ANOVA, as descried in Materials and Methods. ** P < 0.01 vs. 0 h of 27OHChol; * P < 0.05 vs. 0 h of 27OHChol. Statistical differences between treatment with 27OHChol and 27OHChol+Dex at the indicated time periods were evaluated by the Student’s paired t-test. ## P < 0.01 vs. 27OHChol+Dex at 6 h; # P < 0.05 vs. 27OHChol+Dex at 9 h. (B) Whole cell extracts were isolated from the cells and subjected to immunoblotting for p65, phosphorylated p65 and β-actin. Data are representative of three independent experiments.

The inducible transcription factor NF-κB plays a central role in regulation of inflammatory genes [22]. Therefore, we investigated the effects of 27OHChol and dexamethasone on expression of the NF-κB p65 subunit. Transcript levels of the p65 gene were elevated by 1.28-, 2.04-, and 1.77-folds after incubation for 3 h, 6 h, and 9 h with 27OHChol, but these increases were reduced to 0.94-, 0.46-, and 0.95-folds by treatment with dexamethasone, respectively (Fig 5A). We also determined whether dexamethasone affected expression of p65 protein (Fig 5B). The amount of total p65 protein increased in proportion to incubation periods with 27OHChol for up to 9 h, but the expression was inhibited by treatment with dexamethasone. 27OHChol and dexamethasone affected phosphorylation of p65 in a similar fashion as that of expression of total p65 protein (Fig 5B). Taken together, these results indicate that dexamethasone reduces expression of the p65 subunit at the transcript and protein levels.

## Discussion

Monocytes migrate into the arterial wall during progression of atherosclerosis and migrated monocytes differentiate into macrophages, which is activated and secrete inflammatory mediators [23]. This migration is orchestrated by chemokine CCL2, which triggers firm adhesion of monocytes to the endothelium and directs their migration into intima [24]. Administration of dexamethasone reduces the number of macrophages in the aortas of cholesterol-fed-rabbits [5, 6]. Therefore, we determined the inhibitory effects of dexamethasone on 27OHChol-induced activation by measuring CCL2. Treatment with dexamethasone resulted in impaired production of CCL2, as well as migration of monocytic cells. We believe that these results may explain the observation of reduced macrophages in the aortas of rabbits fed a high-cholesterol diet.

Infection with gram-negative bacteria such as *Porphyromonas gingivalis* leads to accelerated development of atherosclerotic lesions in mice after a high-cholesterol diet, and administration of LPS to ApoE-deficient mice results in aggravation of inflammation [25, 26]. LPS is recognized by CD14, which transfers LPS to toll-like receptor 4, initiating an inflammatory responses by enhancing the production of chemokine, including CCL2 [27]. We demonstrated downregulation of mCD14 and sCD14 and inhibition of LPS-induced superinduction of CCL2 in the presence of dexamethasone. These results are in line with Bhattacharyya‘s finding that LPS-mediated expression of inflammatory molecules is inhibited by treatment with dexamethasone [28]. In sum, these findings suggest that dexamethasone inhibits further activation of macrophages by microbial pathogens under an environment rich in oxygenated cholesterol derivatives via downregulation of CD14 molecules at the protein level because dexamethasone did not affect transcription of the CD14 gene.

Activated macrophages show dysregulated synthesis and secretion of matrix metalloproteases (MMPs) [29]. Among MMPs, expression of MMP-9 is linked to macrophage recruitment *in vivo* because deficiency in MMP-9 results in reduced macrophage infiltration in ApoE-knockout mice [30]. In agreement with the results of a previous study [30], MMP-9 production was substantially reduced in concurrence with decreased migration of monocytic cells. The reduced production of MMP-9 is in line with reports that intravenous administration with glucocorticoid results in markedly reduced levels of MMP-9 in patients [31, 32]. Though we demonstrated inhibitory effects of dexamethasone on MMP-9 expression induced by 27OHChol at the mRNA and protein levels, it is possible that dexamethasone downregulates MMP-9 activity at the post-translational level because it can reduce protease activity by increasing tissue inhibitor of metalloproteinase-1 (TIMP-1) [33].

NF-κB is composed of homo- and heterodimeric complexes of members of the Rel family. The most common and best-characterized form of NF-κB is the p65/p50 heterodimer. Upon phosphorylation at the serine residues, the NF-κB dimer translocates to the nucleus where it induces expression of a variety of genes involved in inflammation [22]. Of the two subunits, the p65 subunit is responsible for the strong transcription activating potential of NF-κB [34]. The results of enhanced expression and phosphorylation of the p65 subunit mean that 27OHChol will potentiate transcriptional activity of NF-κB, which is in agreement with the results of a study that reported increased phosphorylation and activation of the subunit in the presence of oxygenated cholesterol derivatives [35]. Moreover, a decreased level of the p65 subunit coincided with those of inflammatory mediators after dexamethasone treatment. Taken together, these results suggest that NF-κB activated in the presence of 27OHChol is likely to play a significant role in inflammation in diseases in which oxygenated cholesterol molecules are involved in their pathogenesis.

In this study, we report new pharmacological effects of dexamethasone; namely, inhibition of monocytic cells activation induced by 27OHChol. We think that the inhibitory effects contribute to anti-inflammatory activity of the drug in a milieu rich in oxygenated cholesterol molecules. In contrast to experimental animal models of atherosclerosis, cardiovascular risk factors are aggravated following high doses and long-term administration of glucocorticoids in clinical studies, which increases cardiovascular events. Therefore, local delivery of dexamethasone by using nanoparticles targeting atherosclerotic lesions may be used as a therapeutic approach against inflammation caused by oxygenated cholesterol molecules in atherosclerosis.

## Supporting information

S1 Fig Effect of dexamethasone (Dex) on viability of THP-1 cells. Serum-starved THP-1 cells were treated for 48 h with indicated amount of Dex in the presence of the 27OHChol. Cell viability was determined by Trypan blue exclusion test. Data are expressed as the means ± SD (n = 3 replicates for each group).
(TIF)

S2 Fig Effect of dexamethasone (Dex) on levels of CD14 transcripts. Serum-starved THP-1 cells were treated with indicated amount of Dex in the presence of the 27OHChol for 48 h. Levels of CD14 transcripts were assessed by real-time PCR. The y-axis values represent the increases of CD14 mRNA levels normalized to GAPDH levels, relative to that of the non-treated THP-1 cells (control). Data are expressed as the means ± SD (n = 3 replicates for each group).
(TIF)

## Abbreviations

27OHChol: 27-hydroxycholesterol
LPS: lipopolysaccharide

## References

[1] Boumpas DT, Chrousos GP, Wilder RL, Cupps TR, Balow JE. Glucocorticoid therapy for immune-mediated diseases: basic and clinical correlates. Ann Intern Med. 1993;119(12):1198–208. . 8239251 10.7326/0003-4819-119-12-199312150-00007

[2] Nashel DJ. Is atherosclerosis a complication of long-term corticosteroid treatment? Am J Med. 1986;80(5):925–9. . 3518440 10.1016/0002-9343(86)90639-x

[3] Schacke H, Docke WD, Asadullah K. Mechanisms involved in the side effects of glucocorticoids. Pharmacol Ther. 2002;96(1):23–43. . 12441176 10.1016/s0163-7258(02)00297-8

[4] Sholter DE, Armstrong PW. Adverse effects of corticosteroids on the cardiovascular system. Can J Cardiol. 2000;16(4):505–11. . 10787466

[5] Asai K, Funaki C, Hayashi T, Yamada K, Naito M, Kuzuya M, et al Dexamethasone-induced suppression of aortic atherosclerosis in cholesterol-fed rabbits. Possible mechanisms. Arterioscler Thromb Vasc Biol. 1993;13(6):892–9. . 8499410 10.1161/01.atv.13.6.892

[6] Naito M, Yasue M, Asai K, Yamada K, Hayashi T, Kuzuya M, et al Effects of dexamethasone on experimental atherosclerosis in cholesterol-fed rabbits. J Nutr Sci Vitaminol (Tokyo). 1992;38(3):255–64. . 1453236 10.3177/jnsv.38.255

[7] Poon M, Gertz SD, Fallon JT, Wiegman P, Berman JW, Sarembock IJ, et al Dexamethasone inhibits macrophage accumulation after balloon arterial injury in cholesterol fed rabbits. Atherosclerosis. 2001;155(2):371–80. . 11254907 10.1016/s0021-9150(00)00605-5

[8] Lemaire S, Lizard G, Monier S, Miguet C, Gueldry S, Volot F, et al Different patterns of IL-1beta secretion, adhesion molecule expression and apoptosis induction in human endothelial cells treated with 7alpha-, 7beta-hydroxycholesterol, or 7-ketocholesterol. FEBS Lett. 1998;440(3):434–9. . 9872417 10.1016/s0014-5793(98)01496-3

[9] Lemaire-Ewing S, Berthier A, Royer MC, Logette E, Corcos L, Bouchot A, et al 7beta-Hydroxycholesterol and 25-hydroxycholesterol-induced interleukin-8 secretion involves a calcium-dependent activation of c-fos via the ERK1/2 signaling pathway in THP-1 cells: oxysterols-induced IL-8 secretion is calcium-dependent. Cell Biol Toxicol. 2009;25(2):127–39. doi: 10.1007/s10565-008-9063-0 . 18317936 10.1007/s10565-008-9063-0

[10] Carpenter KL, Taylor SE, van der Veen C, Williamson BK, Ballantine JA, Mitchinson MJ. Lipids and oxidised lipids in human atherosclerotic lesions at different stages of development. Biochim Biophys Acta. 1995;1256(2):141–50. . 7766691 10.1016/0005-2760(94)00247-v

[11] Guyton JR, Klemp KF. Development of the atherosclerotic core region. Chemical and ultrastructural analysis of microdissected atherosclerotic lesions from human aorta. Arterioscler Thromb Vasc Biol. 1994;14(8):1305–14. . 8049192 10.1161/01.atv.14.8.1305

[12] Brown AJ, Jessup W. Oxysterols and atherosclerosis. Atherosclerosis. 1999;142(1):1–28. . 9920502 10.1016/s0021-9150(98)00196-8

[13] Umetani M, Ghosh P, Ishikawa T, Umetani J, Ahmed M, Mineo C, et al The cholesterol metabolite 27-hydroxycholesterol promotes atherosclerosis via proinflammatory processes mediated by estrogen receptor alpha. Cell Metab. 2014;20(1):172–82. doi: 10.1016/j.cmet.2014.05.013 ; PubMed Central PMCID: PMCPMC4098728. 24954418 10.1016/j.cmet.2014.05.013 PMC4098728

[14] Heo W, Kim SM, Eo SK, Rhim BY, Kim K. FSL-1, a Toll-like Receptor 2/6 Agonist, Induces Expression of Interleukin-1alpha in the Presence of 27-hydroxycholesterol. Korean J Physiol Pharmacol. 2014;18(6):475–80. doi: 10.4196/kjpp.2014.18.6.475 ; PubMed Central PMCID: PMCPMC4296036. 25598661 10.4196/kjpp.2014.18.6.475 PMC4296036

[15] Kim SM, Kim BY, Lee SA, Eo SK, Yun Y, Kim CD, et al 27-Hydroxycholesterol and 7alpha-hydroxycholesterol trigger a sequence of events leading to migration of CCR5-expressing Th1 lymphocytes. Toxicol Appl Pharmacol. 2014;274(3):462–70. doi: 10.1016/j.taap.2013.12.007 . 24370436 10.1016/j.taap.2013.12.007

[16] Kim SM, Lee SA, Kim BY, Bae SS, Eo SK, Kim K. 27-Hydroxycholesterol induces recruitment of monocytic cells by enhancing CCL2 production. Biochem Biophys Res Commun. 2013;442(3–4):159–64. doi: 10.1016/j.bbrc.2013.11.052 . 24269812 10.1016/j.bbrc.2013.11.052

[17] Lemaire-Ewing S, Prunet C, Montange T, Vejux A, Berthier A, Bessede G, et al Comparison of the cytotoxic, pro-oxidant and pro-inflammatory characteristics of different oxysterols. Cell Biol Toxicol. 2005;21(2):97–114. doi: 10.1007/s10565-005-0141-2 . 16142584 10.1007/s10565-005-0141-2

[18] Kim SM, Kim BY, Eo SK, Kim CD, Kim K. 27-Hydroxycholesterol up-regulates CD14 and predisposes monocytic cells to superproduction of CCL2 in response to lipopolysaccharide. Biochim Biophys Acta. 2015;1852(3):442–50. doi: 10.1016/j.bbadis.2014.12.003 . 25497142 10.1016/j.bbadis.2014.12.003

[19] Martin TR, Mongovin SM, Tobias PS, Mathison JC, Moriarty AM, Leturcq DJ, et al The CD14 differentiation antigen mediates the development of endotoxin responsiveness during differentiation of mononuclear phagocytes. J Leukoc Biol. 1994;56(1):1–9. . 7517989 10.1002/jlb.56.1.1

[20] Hailman E, Vasselon T, Kelley M, Busse LA, Hu MC, Lichenstein HS, et al Stimulation of macrophages and neutrophils by complexes of lipopolysaccharide and soluble CD14. J Immunol. 1996;156(11):4384–90. . 8666811

[21] Senft AP, Korfhagen TR, Whitsett JA, Shapiro SD, LeVine AM. Surfactant protein-D regulates soluble CD14 through matrix metalloproteinase-12. J Immunol. 2005;174(8):4953–9. . 15814723 10.4049/jimmunol.174.8.4953

[22] Christian F, Smith EL, Carmody RJ. The Regulation of NF-kappaB Subunits by Phosphorylation. Cells. 2016;5(1). doi: 10.3390/cells5010012 ; PubMed Central PMCID: PMCPMC4810097. 26999213 10.3390/cells5010012 PMC4810097

[23] Andersson J, Libby P, Hansson GK. Adaptive immunity and atherosclerosis. Clin Immunol. 2010;134(1):33–46. doi: 10.1016/j.clim.2009.07.002 . 19635683 10.1016/j.clim.2009.07.002

[24] Libby P, Ridker PM, Maseri A. Inflammation and atherosclerosis. Circulation. 2002;105(9):1135–43. . 11877368 10.1161/hc0902.104353

[25] Li L, Messas E, Batista EL Jr., Levine RA, Amar S. Porphyromonas gingivalis infection accelerates the progression of atherosclerosis in a heterozygous apolipoprotein E-deficient murine model. Circulation. 2002;105(7):861–7. . 11854128 10.1161/hc0702.104178

[26] Ostos MA, Recalde D, Zakin MM, Scott-Algara D. Implication of natural killer T cells in atherosclerosis development during a LPS-induced chronic inflammation. FEBS Lett. 2002;519(1–3):23–9. . 12023012 10.1016/s0014-5793(02)02692-3

[27] Kielian TL, Blecha F. CD14 and other recognition molecules for lipopolysaccharide: a review. Immunopharmacology. 1995;29(3):187–205. . 7542643 10.1016/0162-3109(95)00003-c

[28] Bhattacharyya S, Brown DE, Brewer JA, Vogt SK, Muglia LJ. Macrophage glucocorticoid receptors regulate Toll-like receptor 4-mediated inflammatory responses by selective inhibition of p38 MAP kinase. Blood. 2007;109(10):4313–9. doi: 10.1182/blood-2006-10-048215 ; PubMed Central PMCID: PMCPMC1885507. 17255352 10.1182/blood-2006-10-048215 PMC1885507

[29] Khokha R, Murthy A, Weiss A. Metalloproteinases and their natural inhibitors in inflammation and immunity. Nat Rev Immunol. 2013;13(9):649–65. doi: 10.1038/nri3499 . 23969736 10.1038/nri3499

[30] Luttun A, Lutgens E, Manderveld A, Maris K, Collen D, Carmeliet P, et al Loss of matrix metalloproteinase-9 or matrix metalloproteinase-12 protects apolipoprotein E-deficient mice against atherosclerotic media destruction but differentially affects plaque growth. Circulation. 2004;109(11):1408–14. doi: 10.1161/01.CIR.0000121728.14930.DE . 14993123 10.1161/01.CIR.0000121728.14930.DE

[31] Burnham JA, Wright RR, Dreisbach J, Murray RS. The effect of high-dose steroids on MRI gadolinium enhancement in acute demyelinating lesions. Neurology. 1991;41(9):1349–54. . 1891079 10.1212/wnl.41.9.1349

[32] Rosenberg GA, Dencoff JE, Correa N Jr., Reiners M, Ford CC. Effect of steroids on CSF matrix metalloproteinases in multiple sclerosis: relation to blood-brain barrier injury. Neurology. 1996;46(6):1626–32. . 8649561 10.1212/wnl.46.6.1626

[33] Forster C, Kahles T, Kietz S, Drenckhahn D. Dexamethasone induces the expression of metalloproteinase inhibitor TIMP-1 in the murine cerebral vascular endothelial cell line cEND. J Physiol. 2007;580(Pt.3):937–49. doi: 10.1113/jphysiol.2007.129007 ; PubMed Central PMCID: PMCPMC2075456. 17317742 10.1113/jphysiol.2007.129007 PMC2075456

[34] Schmitz ML, Baeuerle PA. The p65 subunit is responsible for the strong transcription activating potential of NF-kappa B. EMBO J. 1991;10(12):3805–17. ; PubMed Central PMCID: PMCPMC453117. 1935902 10.1002/j.1460-2075.1991.tb04950.x PMC453117

[35] Aye IL, Waddell BJ, Mark PJ, Keelan JA. Oxysterols exert proinflammatory effects in placental trophoblasts via TLR4-dependent, cholesterol-sensitive activation of NF-kappaB. Mol Hum Reprod. 2012;18(7):341–53. doi: 10.1093/molehr/gas001 . 22238372 10.1093/molehr/gas001

