# Supplementary figures and images for: Dexamethasone inhibits activation of monocytes/macrophages in a milieu rich in 27-oxygenated cholesterol
Source: PLoS One. 2017 Dec 13;12(12):e0189643. doi: 10.1371/journal.pone.0189643 (PMC5728574; doi:10.1371/journal.pone.0189643)

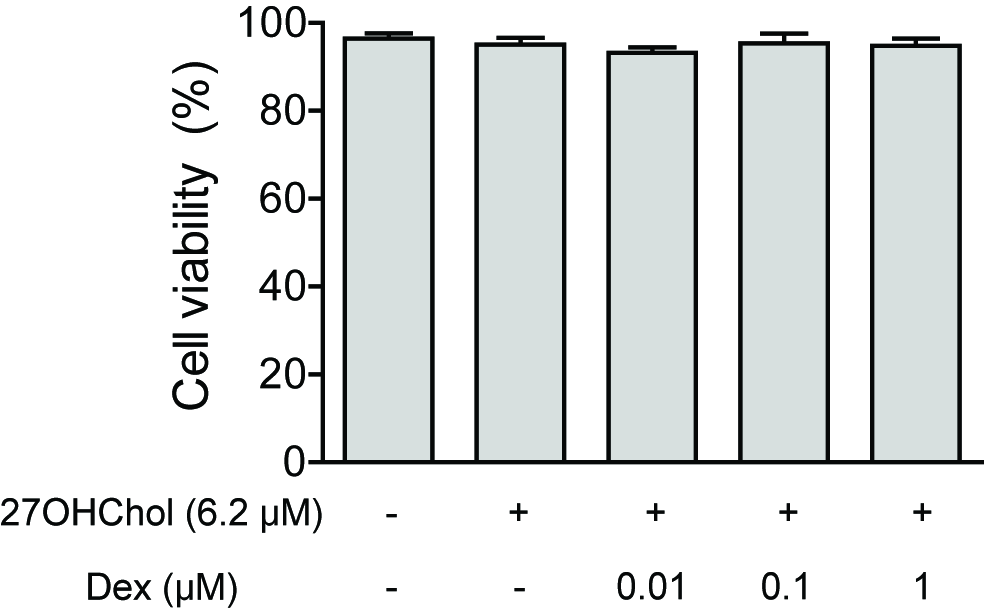

Supplement: S1 Fig — Serum-starved THP-1 cells were treated for 48 h with indicated amount of Dex in the presence of the 27OHChol. Cell viability was determined by Trypan blue exclusion test. Data are expressed as the means ± SD (n = 3 replicates for each group). (TIF) [file pone.0189643.s001.tif]

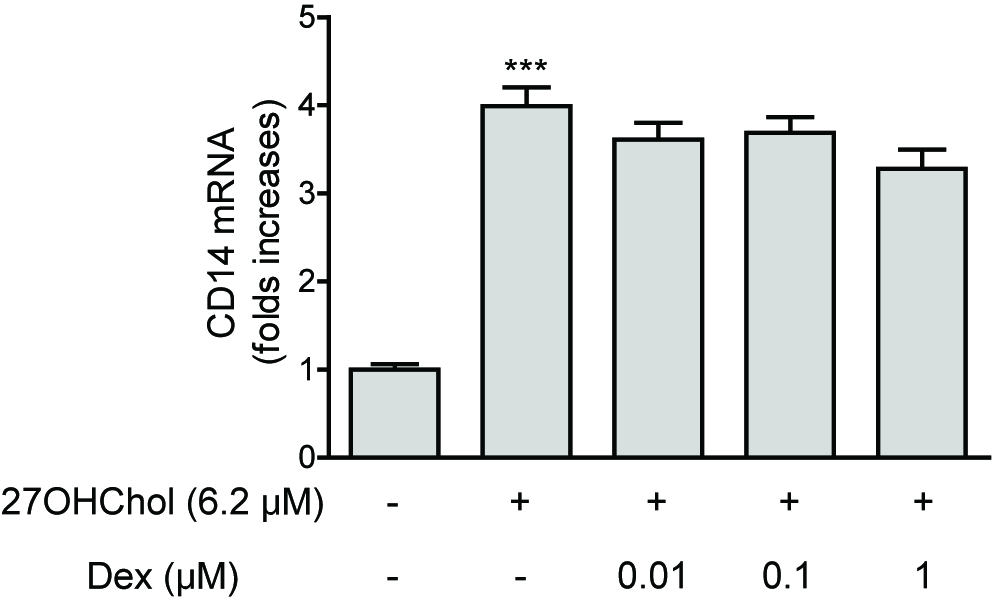

Supplement: S2 Fig — Serum-starved THP-1 cells were treated with indicated amount of Dex in the presence of the 27OHChol for 48 h. Levels of CD14 transcripts were assessed by real-time PCR. The y-axis values represent the increases of CD14 mRNA levels normalized to GAPDH levels, relative to that of the non-treated THP-1 cells (control). Data are expressed as the means ± SD (n = 3 replicates for each group). (TIF) [file pone.0189643.s002.tif]
